# Immunoproteomic Approach of Extracellular Antigens From *Paracoccidioides* Species Reveals Exclusive B-Cell Epitopes

**DOI:** 10.3389/fmicb.2019.02968

**Published:** 2020-01-28

**Authors:** André Luís Elias Moreira, Milton Adriano Pelli Oliveira, Lana O’Hara Souza Silva, Moisés Morais Inácio, Alexandre Melo Bailão, Juliana Alves Parente-Rocha, Vanessa Rafaela Milhomem Cruz-Leite, Juliano Domiraci Paccez, Célia Maria de Almeida Soares, Simone Schneider Weber, Clayton Luiz Borges

**Affiliations:** ^1^Laboratório de Biologia Molecular, Instituto de Ciências Biológicas, Universidade Federal de Goiás, Goiânia, Brazil; ^2^Laboratório de Citocinas, Instituto de Patologia Tropical e Saúde Pública, Universidade Federal de Goiás, Goiânia, Brazil; ^3^Laboratório de Biociência, Faculdade de Ciências Farmacêuticas, Alimentos e Nutrição, Universidade Federal de Mato Grosso do Sul, Campo Grande, Brazil; ^4^Instituto de Ciências Exatas e Tecnologia, Universidade Federal do Amazonas, Itacoatiara, Brazil

**Keywords:** *Paracoccidioides* spp., antigens secreted, epitopes, diagnostic, mass spectrometry

## Abstract

Fungi of the *Paracoccidioides* genus are the etiological agents of paracoccidioidomycosis (PCM), a systemic mycosis restricted to the countries of Latin America. Currently, the *Paracoccidioides* complex is represented by *Paracoccidioides lutzii*, *Paracoccidioides americana*, *Paracoccidioides brasiliensis*, *Paracoccidioides restrepiensis*, and *Paracoccidioides venezuelensis*. Even with advances in techniques used for diagnosing fungal diseases, high rates of false-positive results for PCM are still presented. Additionally, there is no efficient antigen that can be used to follow up the efficiency of patient treatment. The immunoproteomic is considered a powerful tool for the identification of antigens. In addition, antigens are molecules recognized by the immune system, which make them excellent targets for diagnostic testing of diseases caused by microorganisms. In this vein, we investigated which antigens are secreted by species representing *Paracoccidioides* complex to increase the spectrum of molecules that could be used for future diagnostic tests, patient follow-up, or PCM therapy. To identify the profile of antigens secreted by *Paracoccidioides* spp., immunoproteomic approaches were used combining immunoprecipitation, followed by antigen identification by nanoUPLC-MS^E^-based proteomics. Consequently, it was possible to verify differences in the exoantigen profiles present among the studied species. Through a mass spectrometry approach, it was possible to identify 79 exoantigens in *Paracoccidioides* species. Using bioinformatics tools, two unique exoantigens in *P. lutzii* species were identified, as well as 44 epitopes exclusive to the *Paracoccidioides* complex and 12 unique antigenic sequences that can differentiate between *Paracoccidioides* species. Therefore, these results demonstrate that *Paracoccidioides* species have a range of B-cell epitopes exclusive to the complex as well as specific to each *Paracoccidioides* species. In addition, these analyses allowed us the identification of excellent biomarker candidates for epidemiology screening, diagnosis, patient follow-up, as well as new candidates for PCM therapy.

## Graphical Abstract

Immunoproteome overview of *Paracoccidioides* species.

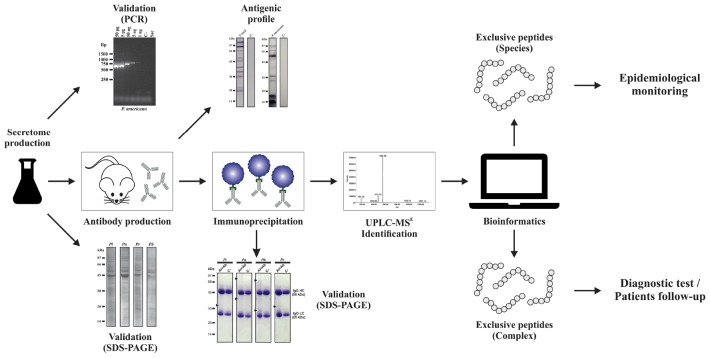

## Introduction

Paracoccidioidomycosis (PCM) is a systemic mycosis restricted to the countries of central and south America and is considered one of the most important endemic mycoses in this region, especially in Brazil (Restrepo et al., 2001). The disease is caused by the fungal species that occupy the genus *Paracoccidioides*: *Paracoccidioides brasiliensis*, *Paracoccidioides lutzii*, *Paracoccidioides americana*, *Paracoccidioides restrepiensis*, and *Paracoccidioides venezuelensis* (Teixeira et al., 2009; Munoz et al., 2016; Turissini et al., 2017).

In the environment, *Paracoccidioides* spp. develop as filamentous structures (hyphae) and when under stress conditions and/or lack of nutrients, the hyphae can produce infectious propagules called conidia. PCM is acquired when an individual inhales conidia or fragments of hyphae that may reach the pulmonary alveoli, giving rise to the yeast form of the fungus, which is considered the parasitic form of *Paracoccidioides* (Wanke and Londero, 1994; Lacaz et al., 2002). Thus, *Paracoccidioides* spp. are characterized as dimorphic and thermally dependent fungi, presenting a saprobiotic mycelial phase and a parasitic yeast phase (Teixeira et al., 2014). Due to these characteristics, when found in ambient or cultured conditions *in vitro*, where temperatures vary around 22–27°C, *Paracoccidioides* spp. grow as mycelium. When the mycelia or conidia are housed in the tissues of the host or incubated *in vitro* at around 36°C, the dimorphic transition to the yeast phase occurs (McEwen et al., 1987; Franco et al., 1989; Brummer et al., 1993; Queiroz-Telles, 1994; Teixeira et al., 2009).

The development of PCM can occur immediately after contact with the fungus or can take years to be triggered. PCM can manifest itself in two clinical forms: acute/subacute (juvenile) and chronic (adult form) (Morejon et al., 2009). The acute and subacute forms of PCM account for 5–25% of cases, being observed in some states of Brazil, such as Maranhão, Goiás, Minas Gerais, Pará, and São Paulo. The chronic form presents in most cases, with a prevalence of 74–96%, manifesting mainly in male adults working in agricultural areas, aged between 30 and 60 years old. The male sex is usually more affected, with a rate of 22 men for every woman (Mendes, 1994; Costa et al., 2013). On the other hand, some patients present clinical manifestations compatible with the acute or subacute form, associated with other clinical manifestations generally observed in the chronic form, making the classification doubtful. In general, these patients demonstrate a very widespread disease with intense suppression of cellular immunity, which is referred to as mixed PCM form. In addition, PCM is a systemic disease whose host response to the infecting agent consists of a chronic granulomatous inflammatory process, which can progress to a fibrous process, characterized as anatomical and functional sequelae in the affected organs, particularly in the lungs (Shikanai-Yasuda et al., 2017, 2018).

Notification of PCM’s cases is not compulsory, and consequently, epidemiological approaches to PCM present certain obstacles. Among these obstacles are the difficulties in recognizing the infection acquired by *Paracoccidioides* species due to the poor capacity of laboratorial diagnosis in endemic areas. These issues lead to the low amount of PCM’s epidemic data, which are obtained from hospital records, epidemiological research reports, case series, and mortality data (Martinez, 2015, 2017). Based on data obtained from care services for PCM patients, the incidence of the disease in endemic areas ranges from one to three cases per 100,000 inhabitants and three to four new cases per 1,000,000 inhabitants per year (Lacaz et al., 2002; Martinez, 2015).

For the diagnosis of PCM, biological materials, such as sputum, biopsy of injured tissues, material from lymph nodes, mucous membranes, and urine can be used, which can be analyzed by optical microscopy. For a long time, immunodiagnosis was not recommended for specific diagnosis of the disease as it presented low specificity, being more commonly indicated for patient follow-up (Shikanai-Yasuda et al., 2006). Despite that, in the *Paracoccidioides’s* literature, there is a robust number of studies that have contributed to the improvement of PCM immunodiagnosis, with the development of serological tests that present high sensitivity and specificity (that reached 90–100%), as revised by Zancope-Oliveira et al. (2014). Even with these advances, the latest “Brazilian Guidelines for the Clinical Management of Paracoccidioidomycosis” still points to the fact that patients with histoplasmosis, aspergillosis, and leishmaniasis may have false-positive results for PCM. Additionally, determining which species of *Paracoccidioides* is the causative agent of the disease is still a restricted approach to some research centers, which limits the expansion of disease epidemiological data (Shikanai-Yasuda et al., 2017). Altogether, these facts point to the need for determination of specific *Paracoccidioides* antigens, for which the immunoproteomics approach is of great value.

The immunoproteomic is considered a powerful tool for the identification of antigens and has been used successfully to verify the response of antibodies to fungal antigens, enabling the identification of possible biomarkers, reactivity profile analysis, as well as characterizing molecules with potential for diagnosis, as demonstrated for several fungal pathogens such as *Aspergillus fumigatus* (Gautam et al., 2007; Virginio et al., 2014), *Candida albicans* (Bar et al., 2012; Lee et al., 2014; Pitarch et al., 2016), *Rhizopus oryzae* (Sircar et al., 2012), *Cryptococcus gattii* (Jobbins et al., 2010), *Coccidioides posadasii* (Tarcha et al., 2006), and *Sporothrix schenckii* (Rodrigues et al., 2015).

To determine specific antigens of *Paracoccidioides* spp., we employed the immunoproteomics approach for identification and characterization of secreted antigens (exoantigens) by representative species of the *Paracoccidioides* complex. With this, it was possible to verify differences in the exoantigen profiles among the species used in this study. Through mass spectrometry tools, it was possible to identify 79 antigens secreted by isolates of *Paracoccidioides*. In addition, bioinformatics tools were used to analyze which antigens are secreted by alternative or classic pathways, as well as which antigenic epitopes are present in these molecules. In addition, we identified which molecules are shared among the isolates under study and which molecules are unique to each representative of the *Paracoccidioides* complex. These results demonstrate that *Paracoccidioides* spp. have several proteins that can be recognized by the human immune system. In addition, these analyses allowed us the identification of new possible molecules for PCM diagnosis, patient follow-ups, and therapy.

## Materials and Methods

### Culture and Maintenance of Microorganisms

Isolates from the *Paracoccidioides* complex, *Pb*01 (*P. lutzii*), *Pb*02 (*P. americana*), *Pb*18 (*P. brasiliensis*), and *Pb*Epm83 (*P. restrepiensis*), were used in this study. The *P. venezuelensis* species was not used during the study because it belongs to a monophyletic population restricted to Venezuela and cases of the disease caused by this species in other regions of Latin America have not been described (Theodoro et al., 2012; Turissini et al., 2017). The yeast phase was maintained *in vitro* at 36°C in a Fava Netto’s semisolid medium for 72 h (Fava-Netto, 1955). The components of the Fava Netto’s culture medium were 0.5% (w/v) yeast extract, 1% (w/v) peptone, 0.5% (w/v) meat extract, 0.3% (w/v) proteose-peptone, 0.5% (w/v) NaCl, 4% (w/v) glucose, 1% (w/v) agar, pH 7.2.

### Production of Extracellular Extracts

Yeasts of each *Paracoccidioides* spp. were transferred to the Fava Netto’s liquid medium and maintained at 36°C under agitation at 120 rpm for 96 h. After this period, cells were washed in phosphate buffered saline (PBS) and counted in Neubauer’s chamber, and the viability was measured using Trypan blue dye. For inoculum, 5 × 10^6^ viable cells/ml were used in liquid medium McVeigh-Morton (MMcM) (Restrepo and Jimenez, 1980) and incubated at 120 rpm for 24 h at 36°C. After incubation, the cells were centrifuged at 2,000 × *g* for 20 min. The supernatant obtained after centrifugation was subjected to vacuum filtration on 0.22-μm membranes (Millipore, United States). The filtered samples were concentrated using a 10-kDa ultrafiltration system (Amicon^®^, Millipore, United States) and then washed three times with PBS (Weber et al., 2012). The quantification of extracellular extracts was determined by the Bradford method (Bradford, 1976).

### Cell Lysis Detection Assay Using Polymerase Chain Reaction (PCR)

The genomic DNA of the isolates was obtained according to the protocol described by Sambrook and Russell (2001). For the PCR reactions, the supernatant of sample secretomes (2 μl) and the genomic DNA obtained were used. The reactions were performed in 40 cycles of 94°C for 30 s, 55°C for 30 s, and 72°C for 2 min. The 681 base pair PCR products were generated using oligonucleotides (sense 5′-GACATGCGTGATATCGACTTG-3′ and antisense 5′-GTGCGCCATGCCATTCT-3′) for the formamidase coding gene (GenBank accession number AY163575). PCR amplicons were detected by 1% (w/v) agarose gel electrophoresis using the GelRed intercalating agent (Biotium^TM^, United States). To verify the sensitivity of PCR, a genomic DNA curve containing five dilutions (50 ng, 5 ng, 50 pg, 5 pg, and 1 pg) was constructed for each *Paracoccidioides* species, as described by Weber et al. (2012).

### One-Dimensional Gel Electrophoresis (1-D SDS-PAGE)

The integrity of the extracellular extracts was verified by 1-D SDS-PAGE (Laemmli, 1970). Thirty micrograms of extracellular extracts from each isolate were prepared with sample buffer [0.2 M Tris–HCl, pH 6.8, 40% (w/v) SDS, 2% (w/v) β-mercaptoethanol, and bromophenol blue traces] and heated in the thermoblock at 100°C for 10 min. Subsequently, the samples were submitted to 12% 1-D SDS-PAGE. As reference, the low-molecular-weight marker (GE Healthcare, United Kingdom) was used. Soon after this step, the gels were stained by Coomassie Blue. ImageQuant 300 (GE Healthcare, United Kingdom) was used to obtain the images.

### Production of Polyclonal Antibodies

Balb/c mice from 8 to 12 weeks old were provided by bioterium of the Instituto de Patologia Tropical e Saúde Pública, da Universidade Federal de Goiás (IPTSP-UFG). The animals were handled according to Conselho Nacional de Controle de Experimentação Animal (CONCEA-MCTIC) and submitted to the Ethics Committee of Comissão de Ética no Uso de Animais (CEUA-UFG) under the registry number 030/2016. Four groups containing five animals each were immunized with extracellular extract samples to induce the production of polyclonal antibodies. Separately, each group was immunized using proteins secreted by *P. lutzii*, *P. americana*, *P. brasiliensis*, and *P. restrepiensis*. For the immunizations, 50 μg of extracellular extracts were used in three doses with intervals of 15 days using the complete Freund’s adjuvant (Sigma-Aldrich, United States) for the first dose and the incomplete Freund’s adjuvant (Sigma-Aldrich, United States) for the second and third immunizations. The negative control was obtained from immunized mice only with Freund’s adjuvant as previously described. After the immunizations, the animals were euthanized, and the whole blood of the animals was collected. Whole blood was incubated at 37°C for 10 min, 4°C for 10 min, and centrifuged at 500 × *g* for 10 min. After centrifugation, the immunized sera containing the polyclonal antibodies were collected.

### Immunoblotting Analysis

Immunoblotting was performed as described by de Oliveira et al. (2018) with some modifications. The extracellular extract samples (30 μg) were submitted to 1-D SDS-PAGE at 12% with subsequent transfer to nitrocellulose membranes. The primary antibodies (1:500) were incubated for 2 h, and the secondary antibody (1:20,000) (alkaline phosphatase-labeled mouse anti-IgG; Sigma-Aldrich, United Kingdom) was incubated for 2 h. The membranes were then washed twice with PBS and once with alkaline phosphatase buffer for 15 min. The chromogenic substrate solution for alkaline phosphatase containing BCIP (5-bromo-4-chloro-3-indolyl phosphate) and NBT (tetrazolium-nitroblue chloride) (Sigma-Aldrich, United Kingdom) was used.

### Coupling of IgG to Protein G-Sepharose and Immunoprecipitation

Protein G-Sepharose^®^ 4B resin (Invitrogen, United States) was used for affinity chromatography. The resin was washed three times with PBS and 1.5 ml of immunized or control serum at a concentration of 1.5 μg/μl. The resin was subsequently added to the affinity columns, incubated at 4°C for 30 min to promote the binding of the antibodies to protein G, which is covalently linked to Sepharose beads. After ligation, the columns were washed three times with PBS. Then, 1.5 mg extracellular extracts from each *Paracoccidioides* species were added to the columns, maintained for 16 h at 4°C for the antigen-antibody binding. Afterward, the columns were washed three times using PBS, then the elution solution buffer [glycine 0.1 M, 0.02% sodium azide (w/v), pH 2.6] was added and incubated for 10 min. Subsequently, the supernatants containing antigens were obtained. The experimental procedure of immunoprecipitation is summarized in Supplementary Figure S1.

### Tryptic Digestion of Exoantigens

Antigens of the *Paracoccidioides* species obtained by immunoprecipitation were subjected to tryptic digestion. Briefly, 10 μl of 50 mM ammonium bicarbonate pH 8.5 were added to 50 μg of the protein extracts and subsequently subjected to tryptic digestion as previously described (Murad and Rech, 2012; Bailao et al., 2014; de Curcio et al., 2017; de Oliveira et al., 2018). Then, 25 μl of 0.2% (w/v) RapiGest SF^TM^ surfactant (Waters Corporation, United Kingdom) was added to the protein extracts and vortexed with subsequent incubation at 80°C for 15 min. After this incubation, 2.5 μl of 100 mM dithiothreitol (GE Healthcare, United States) was added and incubated at 60°C for 30 min. Then, 2.5 μl of 300 mM iodoacetamide (GE Healthcare, United States) was added and maintained at room temperature for 30 min. Subsequently, 10 μl of 50 mM trypsin (Promega, United States) solution was added and incubated for 16 h at 37°C. To precipitate the RapiGest SF^TM^, 10 μl of 5% trifluoroacetic acid (Sigma-Aldrich, United Kingdom) was added and incubated for 90 min at 37°C. The samples were centrifuged at 20,000 × *g* for 30 min at 6°C, and the supernatants were collected. The supernatants were dried by using speed vacuum (Eppendorf, GER). All peptides obtained were resuspended in 80 μl of a solution containing 20 mM ammonium formate and Fosforilase MassPREP^TM^ Digestion Standard (100 fmol/μl for *Pb*Epm83, 400 fmol/μl for *Pb*01, 100 fmol/μl for *Pb*02, and 100 fmol/μl for *Pb*18) as the endogenous standard.

### NanoUPLC-MS^E^ Analysis

After tryptic digestion of the purified exoantigens, the samples were submitted to nanoUPLC-MS^E^. For the chromatographic analyses of peptides, the nanoACQUITY^TM^ M-Class system (Waters Corporation, United Kingdom) was used. For the first dimension, the fragmentation of peptides was performed through a 5-μm UPLC M-Class Peptide BEH C18, 130 Å (300 μm × 50 mm; Waters Corporation, Milford, MA, United States). The fragmented peptides were then submitted to five different acetonitrile/0.1% (v/v) formic acid concentrations (F1, 11.4%; F2, 14.7%; F3, 17.4%; F4, 20.7%; and F5, 50%). Each fraction eluted was trapped in a 5-μm Acquity UPLC M-Class Symmetry C18 Trap Column, 100 Å (180 μm × 20 mm; Waters Corporation, Milford, MA, United States). For the second dimension, for the separation of peptides, an Acquity UPLC M-Class HSS T3 1.8 μm (75 μm × 150 mm) Analytical Column was used. For mass calibration, a solution of 200 fmol/μl of the precursor ion [Glu1]-Fibronopeptide B human (m/z 785.8426) (GFP) (Sigma-Aldrich, St. Louis, MO, United States) was used with a constant flow of 0.5 μl/min, and it was measured every 30 s. The eluted peptides were analyzed by Synapt G1 HDMS^TM^ (Waters Corporation, United Kingdom) mass spectrometer. This device is equipped with nano-electrospray ion source and two mass analyzers, one quadrupole and one time-of-flight (Q-TOF) called nanoESI-Q-TOF (Waters Corporation, United Kingdom). This equipment operates in MS^E^ mode, alternating at 6 V (low power) and 40 V (high power) in each acquisition mode (0.4 s). Three biological replicates were performed for *P. lutzii* (*Pb*01), *P. americana* (*Pb*02), *P. brasiliensis* (*Pb*18), and *P. restrepiensis* (*Pb*Emp83) samples.

### Immunoproteomic NanoUPLC-MS^E^ Data Processing

After nanoUPLC-MS^E^ acquisition, the data processing was performed by using Protein Lynx Global Server version 3.0.2 software (Waters Corporation, United Kingdom). The obtained spectra were compared with sequences deposited in the database of *Pb*01 (*P. lutzii*), *Pb*03 (*P. americana*), *Pb*18 (*P. brasiliensis*), and *Pb*Cnh (*P. restrepiensis*)^1^ to identify the peptides.

### Bioinformatics Analyses

After data processing and protein identification, the identified proteins were subjected to *in silico* analyses. The identified proteins were functionally categorized based on UniProt^2^ database. To predict the subcellular localization of the secreted antigens, WoLF PSORT software^3^ was employed. Expasy software^4^ was used for isoelectric point prediction. For the prediction of proteins secreted by classical and non-classical pathways, SignalP 4.1^5^ and SecretomeP 2.0^6^ were, respectively, used. Regarding SignalP and SecretomeP software, values greater than or equal to 0.5 were considered indicative of secretion. For prediction of linear B-cell epitopes, we used the software BCPREDS^7^ and ABCpred^8^.

To analyze and generate the Venn diagram, we used OrthoVenn software^9^ and Draw Venn Diagram^10^. BLASTp (Basic Local Alignment Search Tool) software^11^ and ClustalX 2.0 and R software^12^ were used for the homology analysis of the identified exoantigens and heat map generation, respectively. ProtScale software^13^ was used for prediction of hydrophilic peptides. The three-dimensional (3-D) modeling of the peptides was performed using I-Tasser^14^ and the figures were visualized by using PyMol version 2.3 software^15^. To verify energy minimization and total structural refinement of 3-D models, the GalaxyWEB^16^ and ModRefiner^17^ servers were used.

## Results

### Monitoring of *Paracoccidioides* Species Cell Integrity Based on PCR Analysis

To verify if cell lysis would influence the profile of proteins secreted by the *P. lutzii* (*Pb*01), *P. americana* (*Pb*02), *P. brasiliensis* (*Pb*18), and *P. restrepiensis* (*Pb*Epm83) species, the PCR technique was used, as described by Weber et al. (2012), with some modifications.

The formamidase gene of *Paracoccidioides* species was analyzed. The sensitivity of the technique was attested to by constructing a standard curve using genomic DNA (samples from 50-ng to 1-pg dilutions) and primers to formamidase gene, generating a 681 base pair amplicon, which was evaluated by agarose gel electrophoresis.

These results demonstrate that this assay was able to amplify extremely low amounts of genomic DNA (1 pg); nevertheless, no amplifications were detected in the supernatant of secretome samples of the isolates under study (Supplementary Figure S2). It was possible to observe that the extracellular extracts did not present PCR-detectable contamination and, accordingly, there are no detectable contaminants from the intracellular compartment since the gene that encodes the formamidase was not amplified in the samples analyzed during the PCR tests (Supplementary Figure S2). This demonstrates that even in the case of any cell lysis, it was undetectable by PCR and thus cannot influence the profile of samples during the proteomics analyses.

### Profile of Extracellular Extracts Evaluated by 1-D SDS-PAGE

Extracellular extracts of *P. lutzii*, *P. americana*, *P. brasiliensis*, and *P. restrepiensis* were analyzed by 12% 1-D SDS-PAGE. After separation, the proteins of isolates depicted a molecular mass distribution between 103 and 16 kDa (Supplementary Figure S3).

High abundance of extracellular protein species by *P. lutzii*, *P. Americana*, and *P. restrepiensis* species was found in the 45-kDa range, demonstrating that the profile of the secreted proteins between the analyzed species presented some similarities (Supplementary Figure S3). Among these, the one with the highest abundance of proteins in the 45-kDa range was the *P. americana* species. The *P. brasiliensis* species did not present high expression of these proteins in the 45-kDa range (Supplementary Figure S3).

### Exoantigen Profiles Assayed by Immunoblotting

**FIGURE 1:**
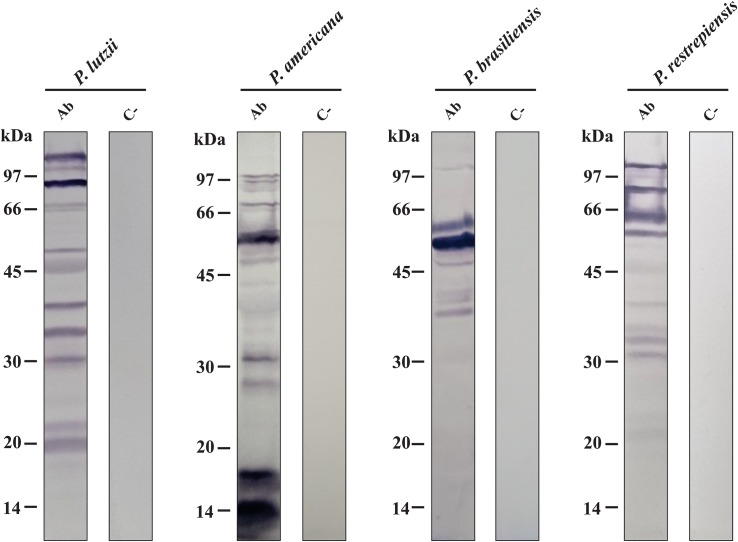
Analysis of the antigenic profile of proteins secreted by *Paracoccidioides* species through immunoblotting. Secretome samples of *Paracoccidioides* species probed against polyclonal antibodies obtained from animals immunized with the secretomes (Ab). Secretome samples of *Paracoccidioides* species probed against serum from mice immunized only with Freund’s adjuvant (C−). kDa, kilodaltons.

Analyses were performed with the objective of obtaining the profile of exoantigens of *Paracoccidioides* species. For this, the extracellular extracts of the *P. lutzii*, *P. americana*, *P. brasiliensis*, and *P. restrepiensis* species were used. Initially, extracellular extracts were subjected to 1-D SDS-PAGE with subsequent transfer to nitrocellulose membranes. Subsequently, immunoblotting assays were performed using antibodies obtained from control and immunized mice (Figure 1).

These results demonstrated that immunoblottings probed with antibodies obtained from immunized mice only with Freund’s adjuvant (controls) did not show any reactivity with the proteins of *Paracoccidioides* species. On the other hand, the serum obtained from mice immunized with secreted protein extracts from *P. lutzii*, *P. americana*, *P. brasiliensis*, and *P. restrepiensis* depicts reactivity against *Paracoccidioides* exoproteomes (Figure 1). In addition, by means of these data, it was possible to observe differences in antigenic profiles among the species representative of the *Paracoccidioides* complex. The antigenic profile of *P. lutzii* showed a wide range of antigenic proteins between 105 and 19 kDa. For the *P. americana* species, a range of antigens between 99 and 14 kDa were presented. Antigens in the range from 99 to 37 kDa were observed in samples from *P. brasiliensis* species. Regarding *P. restrepiensis*, it is possible to observe that the profile of the antigenic proteins varies between 105 and 21 kDa (Figure 1). As a result, it was possible to verify that the secreted protein extracts from *Paracoccidioides* species present antigenic molecules that induce the production of different antibodies.

### Immunoprecipitation of *Paracoccidioides* Exoantigens

**FIGURE 2:**
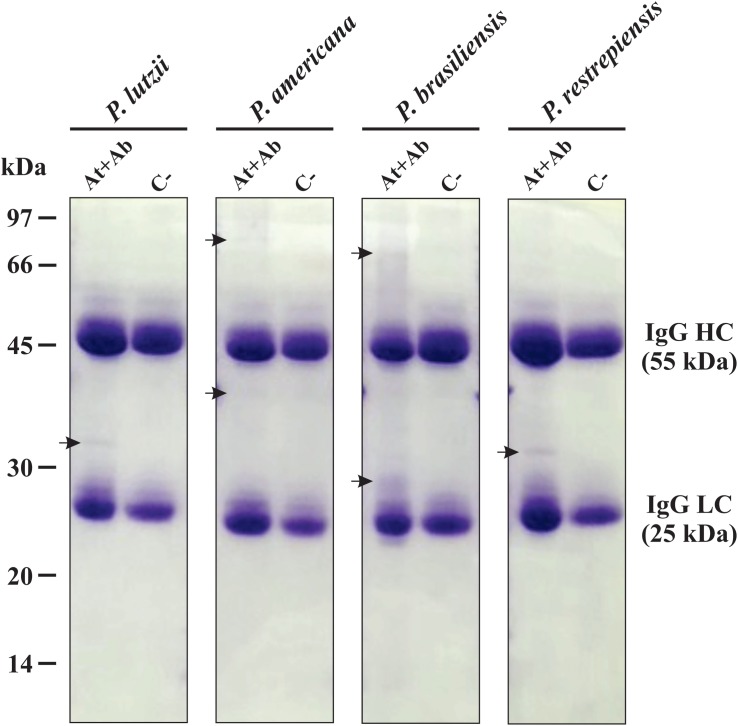
Electrophoretic profile of affinity chromatography. One-dimensional (1-D-SDS-PAGE) gel of proteins eluted from Sepharose beads-IgG. Immunoprecipitation using serum from immunized mice (At + Ab) and negative control (C−). Top row: Isolated representatives of *Paracoccidioides* species (*P. lutzii*, *P. americana*, *P. restrepiensis*, and *P. brasiliensis*). The arrows indicate the presence of exoantigens after purification when compared to the negative control. At, antigen; Ab, antibody; IgG HC, immunoglobulin G heavy chain; IgG LC, immunoglobulin G light chain. Electrophoresis stained with Coomassie blue.

After analyzing the differences in the antigenic profiles between *Paracoccidioides* species, we used immunoprecipitation approach to purify the antibody-reactive exoantigens (Supplementary Figure S1) by using the secretome samples from *P. lutzii*, *P. americana*, *P. brasiliensis*, and *P. restrepiensis* species. The immunoprecipitated samples were separated by 12% 1-D SDS-PAGE and demonstrate the presence of immunoreactive proteins, which proves the antigen-antibody binding (Figure 2). These profile differences are observed according to the molecular weight of the bands of proteins that are visualized in the electrophoresis (Figure 2). However, unlike what was seen regarding the immunized serum, the control sample showed no differences in protein profiles. The absence of immunoreactive proteins in the control sample demonstrates that there was no anti-secretome antibody production (Figure 2).

### Mass Spectrometry Identification of *Paracoccidioides* Species Exoantigens

To identify the immunoprecipitated exoantigens, proteomic strategies were used. Initially, the samples obtained from the *Paracoccidioides* species were digested with trypsin and submitted to nanoUPLC-MS^E^, resulting in the identification of 15 exoantigens for *P. lutzii* (Supplementary Table S1), 14 for *P. americana* (Supplementary Table S2), 17 for *P. brasiliensis* (Supplementary Table S3), and 33 for isolate *P. restrepiensis* (Supplementary Table S4). The immunoprecipitated exoantigens identified in both the immunized and the control serum were removed from the analysis.

**FIGURE 3:**
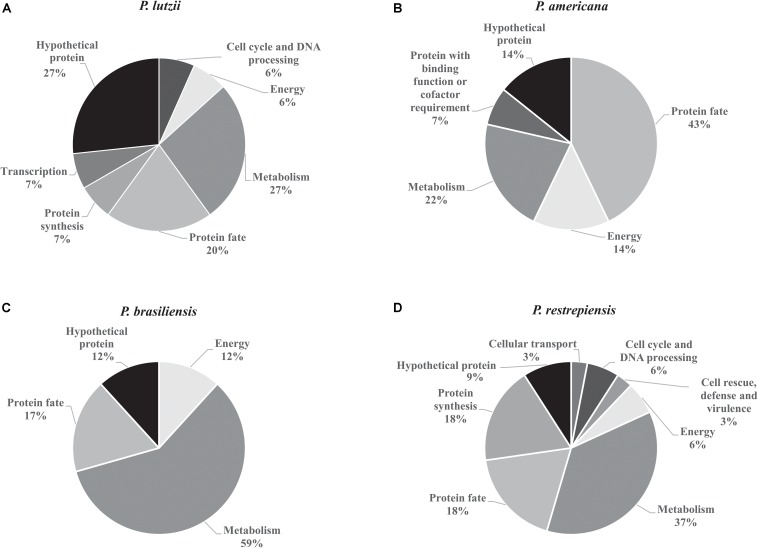
Functional classification of exoantigens identified in *Paracoccidioides* species. **(A)** Functional classification of *P. lutzii* exoantigens. **(B)** Functional classification of exoantigens of *P. americana*. **(C)** Functional classification of *P. brasiliensis* exoantigens. **(D)** Functional classification of exoantigens of *P. restrepiensis*. The classifications of the biological processes were based on UniProt database.

The exoantigens identified in *Paracoccidioides* species were functionally classified in biological processes based on UniProt database. In *P. lutzii*, the most abundant functional class was metabolism (27%), followed by protein fate (20%), energy (6%), transcription (7%), protein synthesis (7%), cell cycle and DNA processing (6%), and hypothetical proteins (27%) (Figure 3A). The most abundant functional classes of *P. americana* after the functional classification were protein fate (43%), metabolism (22%), energy (14%), protein with binding function or cofactor requirement (7%), and hypothetical proteins (14%) (Figure 3B). For *P. brasiliensis*, metabolism (59%) was the most represented functional class, followed by protein fate (17%), energy (12%), and protein synthesis (12%) (Figure 3C). The functional classes of *P. restrepiensis* exoantigens were represented by metabolism (37%), protein fate (18%), protein synthesis (18%), cellular transport (3%), cell cycle and DNA processing (6%), cell rescue, defense, and virulence (3%), energy (6%), and hypothetical proteins (9%) (Figure 3D).

**FIGURE 4:**
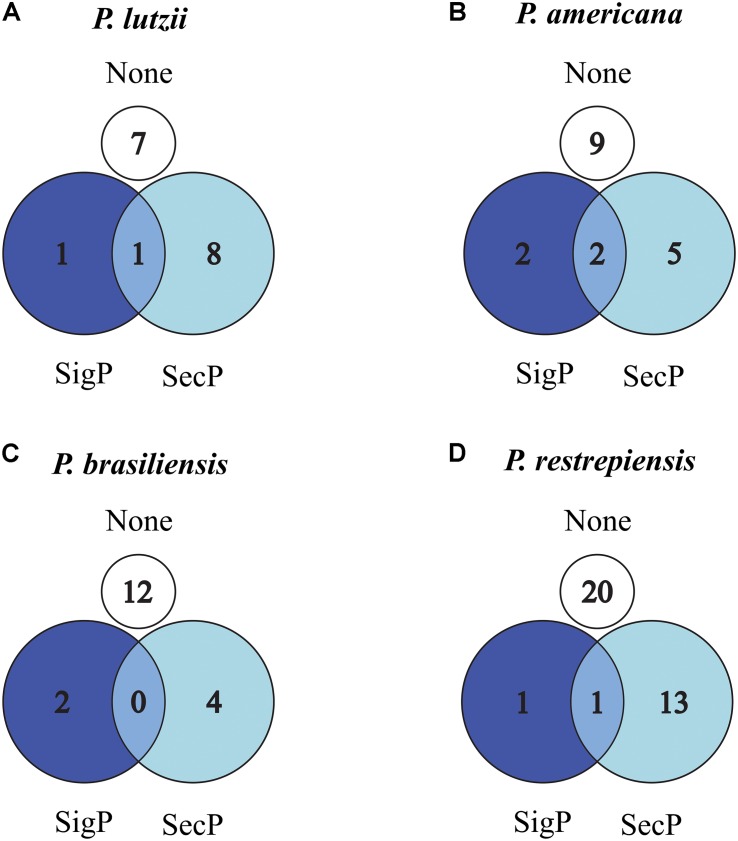
Venn diagram of predicted secreted proteins by classical or alternative route in *Paracoccidioides* species. **(A)** *P. lutzii*, **(B)** *P. americana*, **(C)** *P. brasiliensis*, **(D)** *P. restrepiensis*. Numerical values represented by the number of exoantigens identified. SigP: (SignalP 4.1) prediction of secretion by classical route. SecP: (SecretomeP 2.0) prediction of secretion by alternative routes. None: proteins that have no prediction for secretion in the used algorithms.

To identify signal peptides related to secretion pathways to the extracellular environment by alternative or classic routes, we used SecretomeP and SignalP software, respectively (Supplementary Tables S1–S4). The data obtained by the SecretomeP software revealed that 53.33% (*P. lutzii*), 28.57% (*P. americana*), 23.52% (*P. brasiliensis*), and 39.39% (*P. restrepiensis*) of identified exoantigens were predicted for secretion by non-classics pathways (Figures 4A–D). Using the SignalP software, it was revealed that 6.66, 14.20, 11.76, and 03.03% exoantigens of *P. lutzii, P. americana*, *P. brasiliensis*, and *P. restrepiensis*, respectively, showed signal peptides for secretion by the classical route (Figures 4A–D).

**FIGURE 5:**
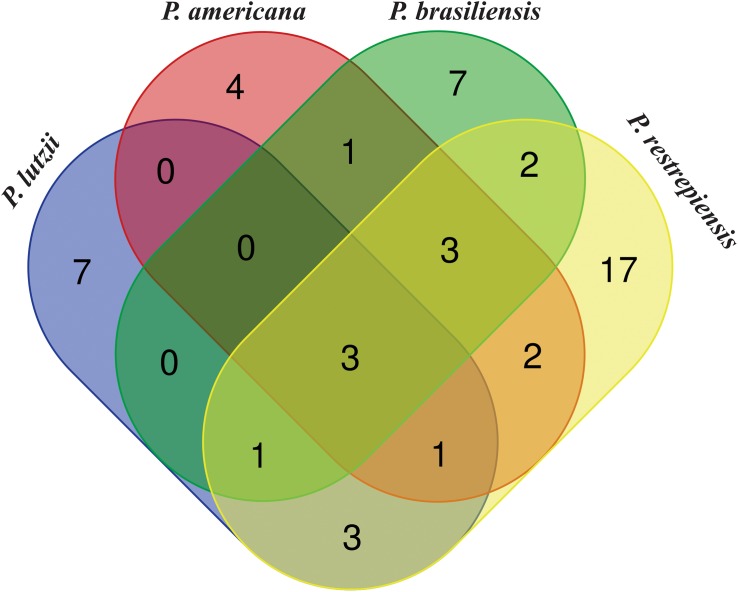
Venn diagram showing common and exclusive exoantigens of *Paracoccidioides* species identified by mass spectrometry. Results are related to exoantigens identified by nanoUPLC-MS^E^. Blue ellipse: *P. lutzii* exoantigens. Pink ellipse: *P. americana* exoantigens. Green ellipse: *P. brasiliensis* exoantigens. Yellow ellipse: *P. restrepiensis*. Crossing the ellipses represents the proteins that were identified in common among *Paracoccidioides* species during immunoproteomics approach.

When comparing the exoantigens identified in *Paracoccidioides* species by software OrthoVenn, seven proteins from *P. lutzii* were exclusives in comparison with other fungus species described in our study. Four, seven, and 17 exoantigens of *P. americana*, *P. brasiliensis*, and *P. restrepiensis* were exclusive to these isolates, respectively (Figure 5 and Supplementary Table S5). The proteins 2-methylcitrate synthase mitochondrial, malate dehydrogenase NAD-dependent, and HSP 70-like protein were identified in all species during the immunoproteomics analyses (Supplementary Table S6). Other proteins, such as endo-1,3(4)-beta-glucanase, dihydrolipoyl dehydrogenase, and glutamate carboxypeptidase, were identified in common between *P. americana*, *P. brasiliensis*, and *P. restrepiensis* species (Supplementary Table S6).

### Non-homologous Exoantigens of *Paracoccidioides* Species

**FIGURE 6:**
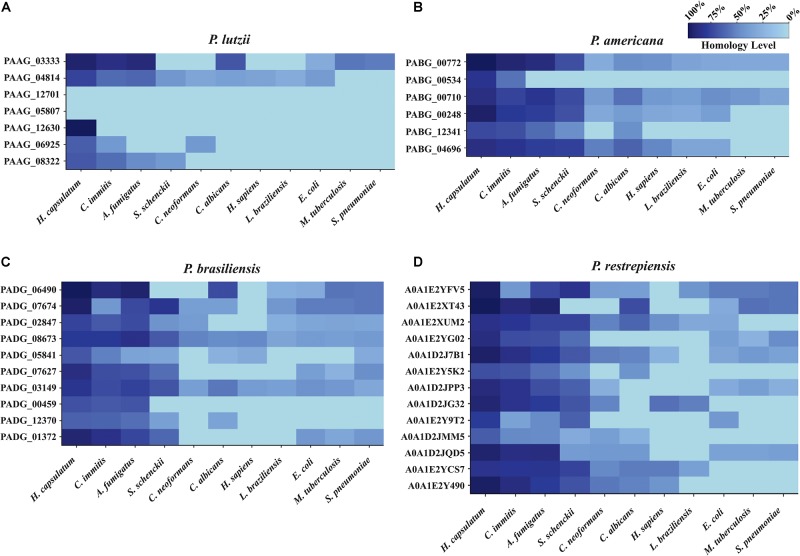
Heat map of exoantigens of *Paracoccidioides* species against other organisms. Heat map showing homology of antigenic molecules of *Paracoccidioides* sp. counts other pathogenic fungi, bacteria, and protozoa. **(A)** *P. lutzii*, **(B)** *P. americana*, **(C)** *P. brasiliensis*, **(D)** *P. restrepiensis*. The homology analysis was performed using the Protein BLAST database, and the graphics were generated using the R software.

A comparative analysis was carried out to verify which molecules were identified as exclusive and conserved among the studied *Paracoccidioides* species. BLASTp-NCBI algorithm was used, and the genome of *P. venezuelensis* was inserted to analyze its exoantigens homology within the *Paracoccidioides* complex. The exoantigens hypothetical protein (PAAG_05807) and glutamate-1-semialdehyde 2,1-aminomutase (PAAG_06925) were exclusive to *P. lutzii* when their homology was compared to the other species of *Paracoccidioides* (Table 1). In addition, the exoantigen hypothetical protein (PAAG_12630) of *P. lutzii* showed low homology to *P. brasiliensis* (72%) and *P. restrepiensis* (76%), but the molecule was not homologous to *P. americana* and *P. venezuelensis*. All other exoantigens analyzed have strong homology between the species of *Paracoccidioides*, demonstrating the presence of these conserved proteins among *Paracoccidioides* species (Supplementary Table S7). In addition, the homology of the *Paracoccidioides* spp. exoantigens was analyzed against proteins of other species and pathogens annotated in the NCBI genome, such as *Homo sapiens* (taxid:9606), *Cryptococcus neoformans* (taxid:5207), *Histoplasma capsulatum* (taxid:5037), *S. schenckii* (taxid:29908), *Coccidioides immitis* (taxid:5501), *C. albicans* (taxid:5476), *A. fumigatus* (taxid:746128), *Mycobacterium tuberculosis* (taxid:1773), *Escherichia coli* (taxid:562), *Leishmania braziliensis* (taxid:5660), and *Streptococcus pneumoniae* (taxid:1313). During the analyses of *P. lutzii* exoantigens, the hypothetical protein (PAAG_12701) and hypothetical protein (PAAG_05807) were not homologous to any of the analyzed species, making these molecules excellent biomarkers to be used for the epidemiological monitoring and diagnosis of PCM caused by *P. lutzii*. The exoantigens hypothetical protein (PAAG_12630) presented homology of 92% to *H. capsulatum*, and glutamate-1-semialdehyde 2,1-aminomutase (PAAG_06925) presented low homology to *H. capsulatum* (62%), *C. immitis* (39%), and *C. neoformans* (39%) (Figure 6A). In *P. americana*, the exoantigen serine protease (PABG_00534) showed homology to *H. capsulatum* (74%) and *C. immitis* (51%). In addition, endo-1,3(4)-beta-glucanase (PABG_12341), although homologous to *H. capsulatum* (66%), *C. immitis* (64%), *A. fumigatus* (54%), *S. schenckii* (42%), and *C. albicans* (41%), presented low homology to the antigens of these species (Figure 6B). In *P. brasiliensis*, the exoantigen ribosome biogenesis protein BMS1 (PADG_00459) was homologous to *H. capsulatum* (66%), *C. immitis* (63%), and *A. fumigatus* (65%). In addition, PTH1 family peptidyl-tRNA hydrolase (PADG_05841) was present in several other pathogens, but exhibited low homology (Figure 6C). For the *P. restrepiensis* species, the exoantigen single-strand binding protein family (A0A1E2Y9T2) presented low homology to *H. capsulatum* (73%), *C. immitis* (33%), *A. fumigatus* (45%), *S. schenckii* (60%), and *E. coli* (39%). Another exoantigen in *P. restrepiensis* that showed low homology was oxidoreductase (A0A1D2JMM5). This molecule was homologous to *H. capsulatum* (61%), *C. immitis* (46%), *A. fumigatus* (45%), *S. schenckii* (28%), *C. neoformans* (35%), and *C. albicans* (24%) (Figure 6D). All comparisons of homology between *Paracoccidioides* spp. and other species of pathogens analyzed during the study are listed in Supplementary Table S8.

**TABLE 1:** Level of homology of the exoantigens of *Paracoccidioides* sp. between the species of the complex.

***P. lutzii*^1,2^**	***P. americana*^3^ (%)**	***P. brasiliensis*^4^ (%)**	***P. restrepiensis*^5^ (%)**	***P. venezuelensis*^6^ (%)**
PAAG_05807 – Hypothetical protein	0	0	0	0
PAAG_12630 – Hypothetical protein	0	72	76	0
PAAG_06925 – Glutamate-1-semialdehyde 2,1-aminomutase	0	0	0	0

*^1^Access number of *P. lutzii* exoantigens. ^2^Exoantigens of *P. lutzii.*^3^Exoantigens of *P. americana* used for homology analysis. ^4^Exoantigens of *P. brasiliensis* used for homology analysis. ^5^Exoantigens of *P. restrepiensis* used for homology analysis. ^6^Exoantigens of *P. venezuelensis* used for homology analysis. Values in % correspond to the homology levels of exoantingens.*

### B-Cell Epitope Prediction of Exoantigens of *Paracoccidioides* Complex

Epitopes are specific regions of an antigen to which an antibody binds. These peptide regions are presented by MHC molecules to T lymphocytes (Abbas et al., 2008). Bioinformatics tools were used during analyses to predict and characterize which B-cell epitopes are present in the exoantigens identified by our immunoproteomics analysis. BCPREDs and ABCpred software were used together to verify if the predicted epitopes would be identified by both tools, leading to confirmation and strengthening of the experimentally obtained data. Initially, the proteins were submitted to BCPREDS software, where the epitopes with the highest score were selected (Supplementary Table S9). Predicted epitopes were also counted for each identified exoantigen, totaling 93 epitopes present in 15 proteins of *P. lutzii* and 158 epitopes present in 14 proteins of *P. americana*. For *P. brasiliensis*, 143 epitopes were predicted in 17 proteins, and there were 232 epitopes in 33 proteins of *P. restrepiensis*. All of these predicted epitopes are listed in Supplementary Table S9.

Subsequently, the epitopes were analyzed using ABCpred software, where the sequences predicted by this tool were selected and compared to the sequences predicted by BCPREDS software. This analysis was performed to verify if these two software tools share the same results, strengthening our experimental data (Supplementary Table S9). Thus, all epitopes predicted by both tools were verified and showed high score values, except for the exoantigens PAAG_12701 of *P. lutzii*, which did not show epitope prediction in any software.

In addition, the amount of predicted epitopes for each identified exoantigen was also evaluated. Interestingly, one of the exoantigens that had the largest number of predicted epitopes was endo-1,3 (4) beta-glucanase with 23 epitopes. This antigen was identified in *P. americana*, *P. brasiliensis*, and *P. restrepiensis* species. Next, aminopeptidase 2 presented 18 epitopes and was identified in *P. americana* and *P. brasiliensis* species. Heat shock proteins such as HSP70 and HSP60 presented 17 and 10 predicted epitopes, respectively, and were identified in all species in this study. The glutamate carboxypeptidase was another exoantigen that showed large numbers of predicted epitopes. A total of 11 epitopes were predicted in this molecule and identified in *P. brasiliensis* and *P. restrepiensis* species. Also, in *P. brasiliensis* and *P. restrepiensis*, the exoantigen dihydrolipoyl dehydrogenase was analyzed and presented nine predicted epitopes (Supplementary Table S9).

### Specific B-Cell Epitopes of *Paracoccidioides* Species

After verifying which exoantigens were homologous to antigenic molecules in other organisms, the homology of the B lymphocyte epitopes predicted by BCPREDS and ABCpred software was verified to identify which antigenic sequences were conserved or not in other organisms. For this, all sequences of the epitopes listed in Supplementary Table S9 were analyzed against homologous epitopes of other important fungi and pathogenic bacteria. Proteins with homology to human molecules were also analyzed to identify and avoid the process of cross-reactivity during future tests. Initially, species such as *C. neoformans*, *H. capsulatum*, *S. schenckii*, *C. immitis*, *C. albicans*, *A. fumigatus*, *M. tuberculosis*, *E. coli*, *S. pneumoniae, L. braziliensis*, and *H. sapiens* were selected. All sequences of proteins homologous to the exoantigens identified during the immunoproteomic were obtained from the UniProt database. ClustalX software was used to perform the alignment and all of the sequences were manually checked. After analysis of all identified epitopes, we selected only those that did not present homology with other analyzed organisms. In addition, the epitope topology in the protein structure is important. To check which epitopes were hydrophilic and can be present in the protein surface, we used ProtScale software, as performed by Ejazi et al. (2018). Thus, we selected only the hydrophilic, non-homologous to other related organisms and exclusive epitopes of the *Paracoccidioides* complex (Table 2). Therefore, five, 11, 10, and 18 epitopes of *P. lutzii*, *P. americana*, *P. brasiliensis*, and *P. restrepiensis*, respectively, were obtained (Table 2). In addition, the homology of all epitopes among *Paracoccidioides* species was evaluated to confirm its presence in all species of this fungus.

**TABLE 2:** B-cell epitopes hydrophilic, non-homologous, and exclusive of *Paracoccidioides* species.

***Paracoccidioides lutzii***	
**Position^1^**	**Access number – Protein name**
	**PAAG_04814 – Nucleic acid-binding protein**
77	VPISRPAPDVRPNETIYIGN
166	SRGSANPTPTRSNEPTRTLF
261	ARDSTMDRLRTAPSGSNETF
	**PAAG_08322 – Hypothetical protein**
33	HIDAREVINPGPVDSPKFFD
264	GTTNPDPSIKDGSSSFASPP
***Paracoccidioides brasiliensis***	
**Position^1^**	**Access number – Protein name**
	**PADG_03149 – Aminopeptidase 2**
162	KIEVASNPSVTVNEDNETAT
	**PADG_07674 – Carbonic anhydrase**
11	STLRTQCTAITTPTRSSINK
43	NKPLPRFPHPCTTRRTISQM
	**PADG_12370 – Endo-1,3(4)-beta-glucanase**
39	TQFPPTGVRLQATRTPGVYH
82	EDHIGLPHPEYPHLDIVPSK
109	CYYKGNWCPGLPHAEENRIS
130	WLLDQSTGAPPKFATVTSSK
163	AAPSATSRSPPRETATVHPS
203	SSMSHQDIFEPIDKGPIPSN
551	IGFGPFDPNTGREATLSNAT
***Paracoccidioides americana***	
**Position^1^**	**Access number – Protein name**
	**PABG_00534 – Serine protease**
81	AYSPIQPPAEVDDPVLVSWA
	**PABG_00710 – Aminopeptidase 2**
162	KIEVASNPSVTVNEDNETAT
	**PABG_04696 – Endonuclease G, mitochondrial**
34	LGTPPPSTRLPPQTTSQPTS
56	IPPHPKITTPDTASPVDPVG
	**PABG_12341 – Endo-1,3(4)-beta-glucanase**
39	TQFPPTGVRLQATRTPGVYH
82	EDHIGLPHPEYPHLDIVPSK
109	CYYKGNWCPGLPHAEENRIS
130	WLLDQSTGAPPKFATVTSSK
163	AAPSATSRSPPRETATAHPS
203	SSMSHQDIFEPIDKGPIPSN
551	IGFGPFDPNTGREATLSNAT
***Paracoccidioides restrepiensis***	
**Position^1^**	**Access number – Protein name**
	**A0A1D2JMM5 – Oxidoreductase**
17	ACQISPQDTTKPPPTVKNRM
55	DVDEVPQPTAEATARTENAD
257	HKEADEHRDQVNVHSPERLC
	**A0A1E2XUM2 – Endonuclease G mitochondrial**
34	LGTPPPSTRLPPQTTSQPTS
	**A0A1E2Y5K2 – Endo-1,3 (4)-beta-glucanase**
39	TQFPPTGVRLQATRTPGVYH
82	EDHIGLPHPEYPHLDIVPSK
109	CYYKGNWCPGLPHAEENRIS
130	WLLDQSTGAPPKFATVTSSK
163	AAPSATSRSPPRETATVHPS
203	SSMSHQDIFEPIDKGPIPSN
551	IGFGPFDPNTGREATLSNAT
	**A0A1E2YCS7 – ATP synthase subunit D**
23	AADSSESEAEGAEEPSIPGG
44	AVTVHSRNEKKARKAIGKLG
80	VINQPDVYRSPSSNTWIIFG
150	EAVEKKDDEEDDGEEVDESG
185	SRKKAIRALKENDNDIVNSI
	**A0A1E2YFV5 – Carbonic anhydrase**
11	STLRTQCTAITTPTRSSINK
43	NKPLPRFPHPCTTRRTISQM

*^1^Position of the epitope in the exoantigen.*

### 3-D Analysis of Exclusive Epitopes of *Paracoccidioides* Species

**FIGURE 7:**
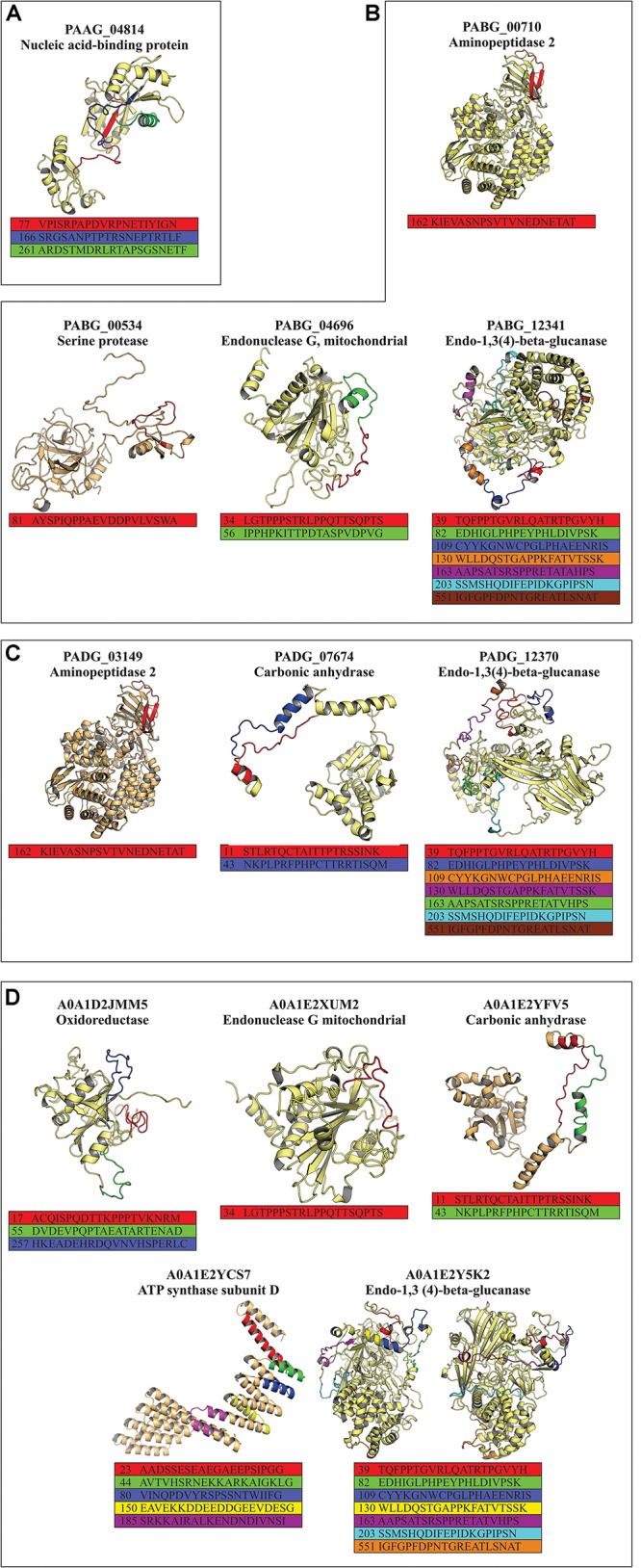
Prediction of the three-dimensional (3-D) model of the exoantigens of *Paracoccidioides* species by I-TASSER. The models were generated using the structure of the exoantigens identified during the immunoproteomics that presented non-homologous epitopes to other pathogenic organisms. The 3-D representation of predicted non-homologous epitopes was performed after structural refinement and energy minimization using ModRefiner and GalaxyWEB software. 3-D view of the non-homologous and hydrophilic epitopes predicted in **(A)** *P. lutzii*, **(B)** *P. americana*, **(C)** *P. brasiliensis*, and **(D)** *P. venezuelensis*.

All exoantigens that showed exclusive epitopes common to all *Paracoccidioides* species (Table 2) had their 3-D structure built by using I-TASSER software and were refined by ModRefiner and GalaxyWEB algorithms. The position of each identified epitope was evaluated in exoantigen models and whether they were present in the surface of the model or not, which possibly facilitates antigen-antibody binding. The epitopes used for the analysis are displayed in Table 2, totaling two, four, three, and five exoantigens for *P. lutzii*, *P. americana*, *P. brasiliensis*, and *P. restrepiensis*, respectively. For *P. lutzii*, the exoantigen nucleic acid-binding protein (PAAG_04814 – 3 epitopes) was evaluated. During the analysis of the 3-D model of this exoantigen, it was possible to verify that one epitope was on the surface (Figure 7A). In *P. americana*, molecular modeling of four exoantigens was made: serine protease (PABG_00534, one epitope), aminopeptidase 2 (PABG_00710, one epitope), endonuclease G, mitochondrial (PABG_04696, two epitopes), and endo-1,3(4)-beta-glucanase (PABG_12341, seven epitopes). After analysis, it was verified that all epitopes analyzed in the exoantigens were present externally to the analyzed models (Figure 7B). Regarding *P. brasiliensis* predicted models, the exoantigens aminopeptidase 2 (PADG_03149, one epitope), carbonic anhydrase (PADG_07674, two epitopes), and endo-1,3(4)-beta-glucanase (PADG_12370, seven epitopes) had their epitopes analyzed in the 3-D models obtained, and it was also possible to verify the presence of the antigens on the surface (Figure 7C). In *P. restrepiensis*, five exoantigens had their epitopes analyzed in generated 3-D models. Thus, the exoantigens oxidoreductase (A0A1D2JMM5, 3 epitopes), endonuclease G mitochondrial (A0A1E2XUM2, one epitope), endo-1,3(4)-beta-glucanase (A0A1E2Y5K2, seven epitopes), ATP synthase subunit D (A0A1E2YCS7, five epitopes), and carbonic anhydrase (A0A1E2YFV5, two epitopes) were analyzed, evidencing that all predicted epitopes were present in the exposed regions in the 3-D models (Figure 7D).

### Exclusive Epitopes of Members of the *Paracoccidioides* Complex

After identification of the exclusive epitopes of *Paracoccidioides*, the level of homology of these epitopes was investigated among species. Thus, the FASTA sequences were obtained of proteins of the *Paracoccidioides* species that were homologous to exoantigens identified during the immunoproteomics assays. For this analysis, homologous molecules of the species *P. lutzii*, *P. americana*, *P. brasiliensis*, *P. restrepiensis*, and *P. venezuelensis* were selected. ClustalX software was used to perform the alignment and the homology of these epitopes in the *Paracoccidioides* complex was manually analyzed. Consequently, non-homologous epitopes to other pathogens were identified that were exclusive to each species of *Paracoccidioides* (Table 3). In *P. lutzii*, seven epitopes from two proteins, hypothetical protein (PAAG_05807) and hypothetical protein (PAAG_12630), were identified (Table 3). In *P. brasiliensis*, three predicted epitopes were exclusive. Two epitopes were present in malate dehydrogenase NAD-dependent (PADG_08054) and one in endo-1,3(4)-beta-glucanase (PADG_12370) (Table 3). For *P. restrepiensis*, two epitopes were identified and belong to ER lumen protein retaining receptor 2 (A0A1E2Y490) (Table 3). Identification of these epitopes needs to be tested to be used during epidemiological monitoring of the disease, supporting the recognition of which species of *Paracoccidioides* is causing PCM.

**TABLE 3:** Exclusive epitopes of each species of *Paracoccidioides* complex.

***Paracoccidioides lutzii***	
**Position^1^**	**Access number – Protein name**
	**PAAG_05807 – Hypothetical protein**
25	NLWRHFNPDTAVVYNHMRSL
70	KNSIRKVGFTPLDNSEPETH
103	SITTPIGQSGHTHPNLTTSW
	**PAAG_12630 – Hypothetical protein**
43	EIGGEMRDTKGARENIQRVK
73	GFRVRGGGEGSEGETVQEGS
94	AAGCGRAKGGKRQGQRGRAK
146	PMGGVDGGTPGPFGKRREDE
***Paracoccidioides brasiliensis***	
**Position^1^**	**Access number – Protein name**
	**PADG_08054 – Malate dehydrogenase NAD-dependent**
7	LGASGGIGQVRGSYMTYNTI
28	KTSSTLWASTPKPNSNKMQP
	**PADG_12370 – Endo-1,3(4)-beta-glucanase**
637	NNYVWMKSDNVNQPDADTPQ
***Paracoccidioides restrepiensis***	
**Position^1^**	**Access number – Protein name**
	**A0A1E2Y490 – ER lumen protein retaining receptor 2**
42	PSDVHIDSPPEDEVVKCLSF
140	RYEFSEVYKAPPYPPPPCIL

*^1^Position of the epitope in the exoantigen.*

## Discussion

In this work, several exoantigens were identified in the representatives of the *Paracoccidioides* complex: *P. lutzii*, *P. americana*, *P. brasiliensis*, and *P. restrepiensis*. These data provide the first large-scale identification of exoantigens of *Paracoccidioides* species. Some of these exoantigens have already been identified and described as antigens of pathogenic fungi, such as formamidase (Borges et al., 2005, 2010), dihydrolipoyl dehydrogenase (Landgraf et al., 2017), HSP60 (Izacc et al., 2001; Cunha et al., 2002), HSP70 (Bromuro et al., 1998; Bisio et al., 2005), serine protease (Parente et al., 2010; Lacerda Pigosso et al., 2017), and isocitrate lyase (Cruz et al., 2011; Silva et al., 2019). This demonstrates the robustness of method used in our immunoproteomics analyses.

Taking into account that our target is the characterization only of secreted antigens, we were able to produce and purify this group of proteins once no DNA from fungal cell lysis was detected in PCR-based assays. Similar results have already been observed in secretome analyses of *Paracoccidioides* sp. (Weber et al., 2012). Subsequently, we verified the profile of secretome obtained, where the molecular weight profiles were found to vary from 16 to 103 kDa, being compatible with the protein profile secreted by *Paracoccidioides* spp. (Weber et al., 2012; de Oliveira et al., 2018), which secreted a range of complex proteins of several sizes. Additionally, it was observed that *Paracoccidioides* species have differences in the exoantigen profiles, with an immunoreactive profile presenting differences between the studied species, although some exoantigens seem to be shared by all species. These antigenic differences may be related to the singular genetic compositions of each species analyzed, as reported (Pigosso et al., 2013; do Amaral et al., 2019). In *Sporothrix* spp., immunoblotting and immunoproteomics approach also have been demonstrating a dissimilar profile between species of complex, highlighting the importance of antigenic molecules as potential biomarkers of serological diagnosis and candidates for vaccine development (Rodrigues et al., 2015).

Currently, immunoprecipitation is a technique used for the purpose of purifying protein extracts, concentrating low-abundance proteins, analyzing protein-protein interactions, posttranslational modification studies, and can also be employed for antigens precipitation (Lund-Johansen et al., 2000). Employing this technique, we found the presence of immunoreactive bands when compared to control samples. Similar results were observed when immunoprecipitation was used for antigen purification with subsequent construction of new diagnostic tests for African trypanosomiasis (Sullivan et al., 2013). In addition, we found that the immunoreactive proteins depict a distinct pattern among the studied species, although some exoantigens were present in all *Paracoccidioides* species, as demonstrated by our proteomics analyses. It can be inferred that these differences can be related to the genetic background, mechanisms involved in the protein expression, as well as the secretion machinery of each *Paracoccidioides* species (Desjardins et al., 2011; Pigosso et al., 2013; de Oliveira et al., 2018; do Amaral et al., 2019; Peres da Silva et al., 2019). Thus, the use of this technique for the purification of antigenic molecules has been shown to be highly effective for obtaining exoantigens from *Paracoccidioides* species.

Additionally, we characterized the *Paracoccidioides* identified antigenic proteins in functional classes. The distribution of secreted proteins in the functional classes was consistent with previous analyses of secretome of *Paracoccidioides* spp. (Weber et al., 2012; de Oliveira et al., 2018) and with proteins secreted in vesicles in other pathogenic fungi (Albuquerque et al., 2008; Rodrigues et al., 2008, 2014; Oliveira et al., 2010). Also, we verified that the protein profile obtained, including mass range and isoelectric point (IP), is consistent with other *Paracoccidioides* sp. secretomes (Weber et al., 2012). Most of the identified antigenic proteins depicted prediction of subcellular localization in the cytoplasm and mitochondria, which is in accordance with described secretomes. These proteins should perform non-classical functions at other cellular locations, being described as moonlighting proteins (Karkowska-Kuleta and Kozik, 2014). The secretion of these molecules seems to be related to virulence and/or host-pathogen interaction of *Paracoccidioides* spp. once it has been demonstrated that secretion of immunogenic molecules in other pathogens is implicated in virulence during host-pathogen interaction (Oliveira et al., 2010; Campbell et al., 2015; de Oliveira et al., 2018).

As expected, the identification of similar exoantigens among *Paracoccidioides* species was confirmed by mass spectrometry analysis. When comparing the identified proteins, some molecules, such as malate dehydrogenase NAD-dependent, HSP70-like protein, and 2-methylcitrate synthase, were present in all species. On the other hand, several exoantigens were identified as unique in the analyzed species. Previous analysis has shown constitutively and differentially expressed molecules between *Paracoccidioides* species (Pigosso et al., 2013; do Amaral et al., 2019). However, analyses of exclusive proteins among the isolates could possibly be used in the differential diagnosis of PCM, as well as epidemiology studies.

Homology analyses were used to identify unique exoantigens of *Paracoccidioides* species. These analyses were initially employed among the exoantigens identified by nanoUPLC-MS^E^, resulting in the identification of exclusive exoantigens to *P. lutzii*. The identification of these unique exoantigens enables its use in the differential diagnosis of PCM caused by *P. lutzii*. Subsequently, all exoantigen sequences were compared to sequences of other fungal and bacterial pathogens to verify the homology of these molecules to other organisms. These homology analyses were also employed on human sequences (Virginio et al., 2014; Rodrigues et al., 2015), aiming to select non-homologous molecules and to avoid possible cross-reactivity in future diagnostic tests. However, a small percentage of the exoantigens analyzed presented low homology, being represented by *P. lutzii* and *P. americana* species. Because homology analysis is performed throughout the exoantigen sequence, we decided to check the homology singly analyzing only the homology of the predicted epitopes. Thus, we identified several exclusive and conserved epitopes in the *Paracoccidioides* complex, since these molecules are candidates for the specific diagnosis of PCM. Similar analyses have already been performed in other works, where linear B-cell epitopes were employed for serological detection of several pathogenic organisms, such as *Borrelia miyamotoiis* (Tokarz et al., 2019), *Toxoplasma gondii* (Gatkowska et al., 2019), *Treponema pallidum* (Ogden et al., 2019), *Leishmania* spp. (Bremer Hinckel et al., 2019; Vale et al., 2019), and several human viruses (Amrun et al., 2019; Mutsvunguma et al., 2019; Yao et al., 2019). This reinforces the importance of these analyses, demonstrating the applications of linear B-cell epitopes in the diagnosis of human pathogenic organisms.

Similar to findings for other fungi, the cell wall of *Paracoccidioides* spp. is composed of a network of glycoproteins and polysaccharides, which act to protect the cell from environmental stressors (De Groot et al., 2005). Recent studies have demonstrated that both the yeast and mycelial phases of *Paracoccidioides* spp. present different amounts of β-(1,3) glucans (Camacho and Nino-Vega, 2017). However, enzymes, such as endo β-(1,3)-glucanase, act by hydrolyzing the β-glucan chains, making these enzymes extremely important because they play a fundamental role in the morphological processes of the fungal cell (Adams, 2004). Endo-1,3(4)-beta-glucanase was found as an antigen secreted in yeasts of *P. americana*, *P. brasiliensis*, and *P. restrepiensis* during our analyses. This enzyme has also been detected as secreted in yeast proteomes of *H. capsulatum* (Garfoot et al., 2017). In addition, the endo-1,3(4)-beta-glucanase showed low homology when compared to other fungi and pathogenic bacteria. Despite the low homology, we decided to investigate the level of homology between the epitopes of this molecule. During analysis, seven specific B-cell epitopes presented as exclusive for the *Paracoccidioides* complex, being excellent candidates for synthesis of new diagnostic tests for PCM.

Interestingly, the carbonic anhydrase (CA) of *Paracoccidioides* spp. was first reported as a secreted antigen in the present study. This is a metalloenzyme and acts by catalyzing the reversible hydration of CO_2_ to generate HCO${}_{3}^{-}$ (Supuran, 2008). Additionally, in *Paracoccidioides* sp., four coding genes for carbonic anhydrase have already been described, leading to the synthesis of CA1, CA2, and CA3 proteins for Class β and CA4 protein for Class α. In addition, this enzyme was positively regulated in *Paracoccidioides* yeast when compared to the mycelium phase of the fungus (Tomazett et al., 2016). In *Stenocarpella macrospora*, CA has already been described in the mitochondrial, cytoplasmic, and extracellular compartments (Elleuche and Poggeler, 2010). CA has been identified as an exoantigen in the current work and has been associated with the extracellular compartment. In addition, CA presented exclusive epitopes, which makes this molecule a potential candidate as a PCM biomarker. Additionally, this exoantigen can be characterized as a moonlighting protein because it has a metabolic function in the cytoplasm and, when exported to the extracellular environment, it is probably related to the virulence of the fungus. In addition, CA has been described and is related to morphogenesis and growth of *Paracoccidioides* sp. and *C. neoformans* (Bahn et al., 2005; Costa et al., 2007) during the infectious processes, suggesting the importance of this molecule during host-pathogen interactions.

Aminopeptidase 2 was identified as an exoantigen during analyses. These molecules are characterized as exopeptidases and act as cleaving proteins in their N-terminal region (Gonzales and Robert-Baudouy, 1996). In *Aspergillus oryzae* mycelium secretome, aminopeptidase was related to collagen degradation processes (Ding et al., 2014). Furthermore, in a recent study, aminopeptidase demonstrated its antigenic potential during the expression of recombinant proteins of *Fasciola hepatica* when probed against antibodies from patients with fascioliasis (Mirzadeh et al., 2018) and against anti-aminopeptidase monoclonal antibodies from *Taenia pisiformis* (Zhang et al., 2018). The antigenicity of this molecule was also attested to in eggs and adult worms of *Schistosoma mansoni* following mouse infection assays (Maggioli et al., 2018). In *Aspergillus terreus* mycelium exoantigens, this molecule showed a high antigenicity when probed against monoclonal antibodies, demonstrating its antigenic profile (Nayak et al., 2011). During analyses, these molecules were identified in *P. americana* and *P. brasiliensis*, where one epitope was characterized as exclusive of the *Paracoccidioides* complex. These characteristics indicate that this molecule may be an excellent candidate to be tested as a potential specific biomarker for *Paracoccidioides* species infections.

Therefore, further analyses are underway to investigate the potential of these molecules as biomarkers, which may be used in the design of a rapid diagnostic test for PCM, in patient treatment follow-ups, as well as to investigate the immunogenic potential of these molecules and their possible use in the therapy of this important neglected human systemic mycosis.

## Conclusion

The differences among exoantigen expression profiles were essential to identify specific biomarkers for each *Paracoccidioides* species. Here, the use of an immunoproteomics approach allowed us the characterization of 15, 14, 17, and 33 exoantigens in *P. lutzii*, *P. americana, P. brasiliensis*, and *P. restrepiensis*, respectively. Additionally, bioinformatics analyses made it possible to perform a series of other characterizations, allowing us to verify the biological functions, homology of exoantigens, epitopes prediction, as well as strengthen the data accuracy. Two exoantigens were identified and described in this work as unique in *P. lutzii*. Also, 44 epitopes exclusive to the *Paracoccidioides* complex were mapped using bioinformatics. Regarding the epitopes that could be used for epidemiological monitoring of the disease, a total of 12 antigenic sequences were identified in exoantigens in the four *Paracoccidioides* species studied. Therefore, these findings demonstrate that *Paracoccidioides* species have a range of epitopes exclusive to the complex, as well as specific to each fungal species. Finally, the current serological tests could be improved further with combinations of two and more synthetic peptides, as well as modifications that can optimize these tests. It is important to highlight that the reactivity of these proposed epitopes is under evaluation and will be tested against clinical samples to provide a new tool for diagnosis, patient follow-ups, and/or PCM therapy.

## Data Availability Statement

The raw data supporting the conclusions of this article will be made available by the authors, without undue reservation, to any qualified researcher.

## Ethics Statement

The animal study was reviewed and approved by the Comissão de Ética no Uso de Animais (CEUA-UFG) under the registry number 030/2016.

## Author Contributions

AM, CB, MO, AB, and CA conceptualized and designed the study. AM, LS, VC-L, and JP worked on data acquisition. AM, CB, AB, SW, JP-R, MI, and CA analyzed and interpreted the data. AM, CB, and JP drafted the manuscript. CA, SW, JP, and CB critically revised the manuscript.

## Conflict of Interest

The authors declare that the research was conducted in the absence of any commercial or financial relationships that could be construed as a potential conflict of interest.

## Footnotes

1 http://www.broadinstitute.org/annotation/genome/paracoccidioides_brasiliensis/Multihome.html

2 http://www.uniprot.org/

3 http://wolfpsort.hgc.jp/

4 https://web.expasy.org/compute_pi/

5 http://www.cbs.dtu.dk/services/SignalP/

6 http://www.cbs.dtu.dk/services/SecretomeP/

7 http://omictools.com/bcpreds-tool

8 http://www.imtech.res.in/raghava/abcpred/

9 www.bioinfogenome.net/OrthoVenn/

10 http://bioinformatics.psb.ugent.be/webtools/Venn/

11 http://blast.ncbi.nlm.nih.gov/

12 http://cran.r-project.org

13 https://web.expasy.org/protscale/

14 http://zhanglab.ccmb.med.umich.edu/I-TASSER/

15 http://pymol.org/2/

16 http://galaxy.seoklab.org/

17 http://zhanglab.ccmb.med.umich.edu/ModRefiner/

## Supplementary Material

FIGURE S1 Affinity chromatography obtained by immunoprecipitation. **(A)** Scheme used for purifying and obtaining the antigens using serum from immunized and control animals. **(B)** Scheme demonstrating the interaction of sepharose bead – G protein bound antibody and antibody antigen interaction.

FIGURE S2 Confirmation by PCR of the extracts secreted by *Paracoccidioides* species. The sensitivity of the PCRs to the formamidase gene were obtained using the genomic DNA of *P. lutzii*, *P. americana*, *P. brasiliensis*, and *P. restrepiensis* in five dilutions ranging from 50 ng to 1 pg (Top panel). C−: Negative control (without genomic DNA). Sec: Cell-free secretome. bp: Base pairs according to the molecular weight marker. The amplicons obtained by PCR were evaluated by 1% agarose gel electrophoresis and stained by GelRed DNA intercalator.

FIGURE S3 Integrity analysis of the proteome profile of the secreted isolates of *Paracoccidioides* species by one-dimensional electrophoresis. Twenty micrograms of samples were separated by one-dimensional electrophoresis (SDS-PAGE) at 12%. *P. lutzii*, *P. americana*, *P. restrepiensis*, and *P. brasiliensis*. kDa, Kilodaltons. Coomassie Blue staining was used to visualize the proteins.

TABLE S1 Identification of exoantigens recognized by serum from animals immunized with *P. lutzii* secretome.

TABLE S2 Identification of exoantigens recognized by serum from animals immunized with *P. americana* secretome.

TABLE S3 Identification of exoantigens recognized by serum from animals immunized with *P. brasiliensis* secretome.

TABLE S4 Identification of exoantigens recognized by serum from animals immunized with *P. restrepiensis* secretome.

TABLE S5 Exoantígens of *Paracoccidioides* species identified as exclusive during immunoproteomic analyzes.

TABLE S6 Exoantígens of *Paracoccidioides* species identified as common during immunoproteomic analyzes.

TABLE S7 Level of homology of the exoantigens of *Paracoccidoides* spp. between the species of the complex.

TABLE S8 Heat map of homology levels of *Paracoccidioides* exoantigens against other organisms.

TABLE S9 *In silico* mapping of B-cell epitopes of all exoantigens identified during the immunoproteome by BCPREDs and ABCpreds.
