# Supplementary figures and images for: Immunoproteomic Approach of Extracellular Antigens From Paracoccidioides Species Reveals Exclusive B-Cell Epitopes
Source: Front Microbiol. 2020 Jan 28;10:2968. doi: 10.3389/fmicb.2019.02968 (PMC7015227; doi:10.3389/fmicb.2019.02968)

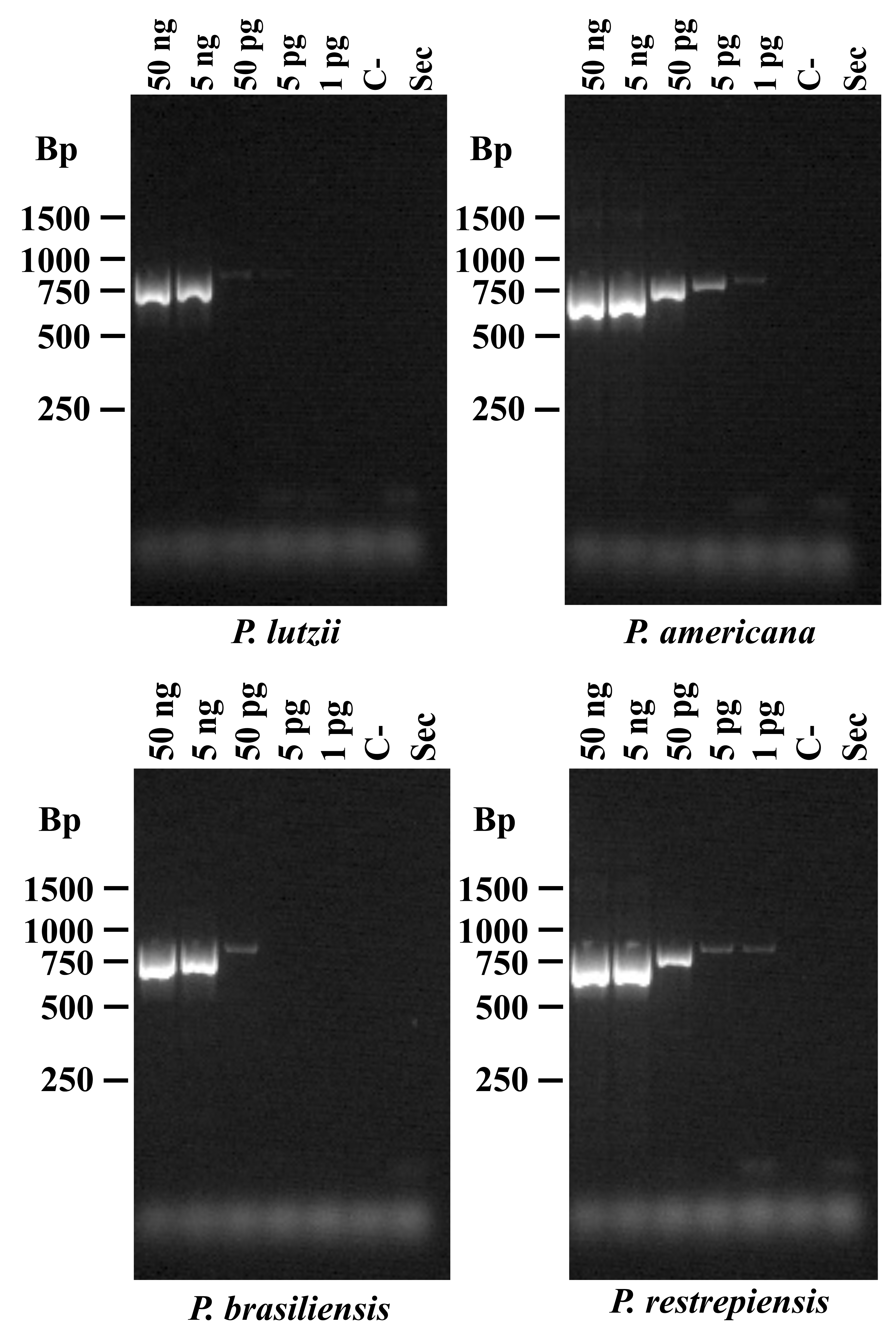

Supplement: FIGURE S2 — Confirmation by PCR of the extracts secreted by Paracoccidioides species. The sensitivity of the PCRs to the formamidase gene were obtained using the genomic DNA of P. lutzii, P. americana, P. brasiliensis, and P. restrepiensis in five dilutions ranging from 50 ng to 1 pg (Top panel). C−: Negative control (without genomic DNA). Sec: Cell-free secretome. bp: Base pairs according to the molecular weight marker. The amplicons obtained by PCR were evaluated by 1% agarose gel electrophoresis and stained by GelRed DNA intercalator. [file Image_2.TIF]

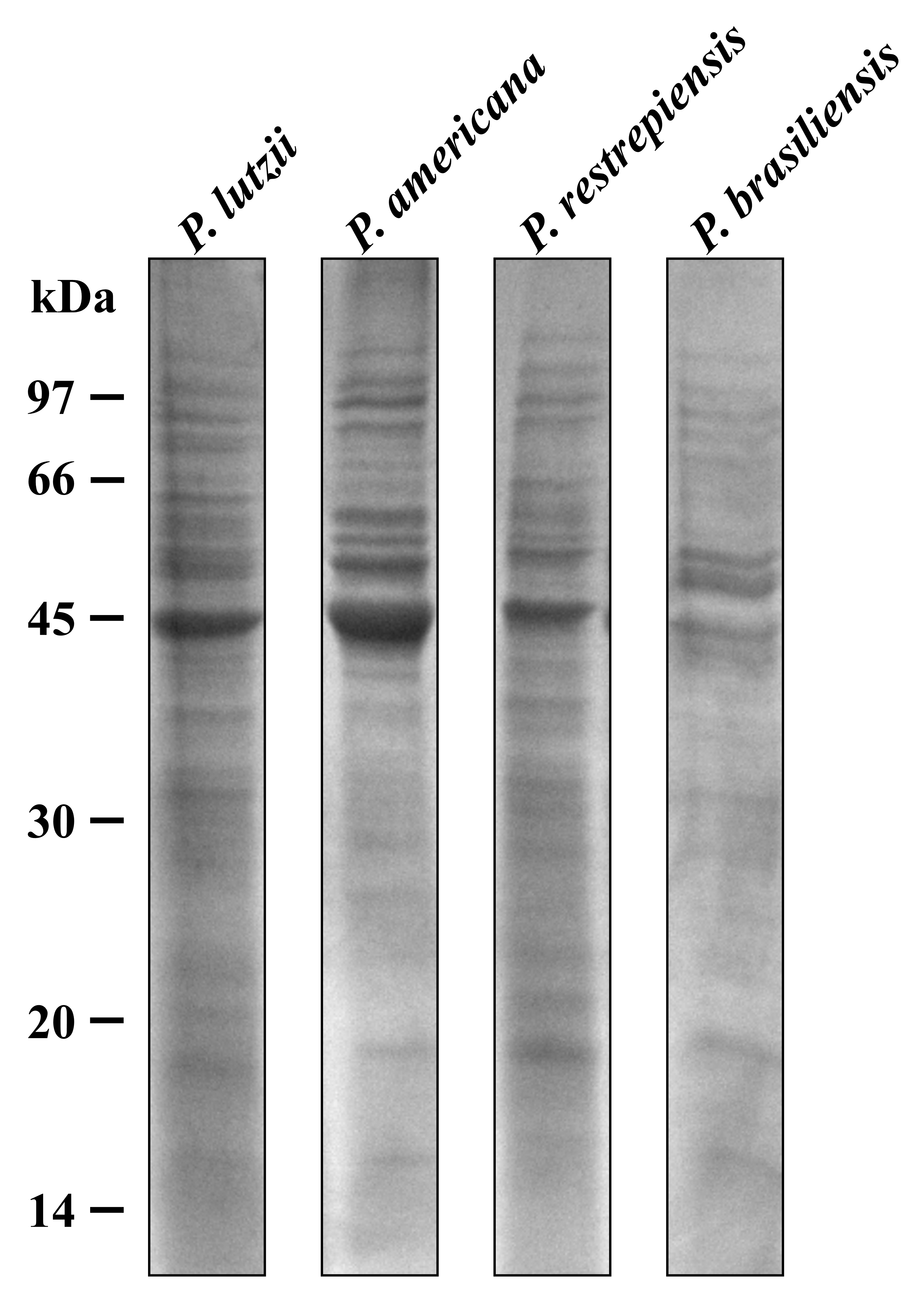

Supplement: FIGURE S3 — Integrity analysis of the proteome profile of the secreted isolates of Paracoccidioides species by one-dimensional electrophoresis. Twenty micrograms of samples were separated by one-dimensional electrophoresis (SDS-PAGE) at 12%. P. lutzii, P. americana, P. restrepiensis, and P. brasiliensis. kDa, Kilodaltons. Coomassie Blue staining was used to visualize the proteins. [file Image_3.TIF]
